# Outbreak of Multidrug-Resistant (MDR) *Acinetobacter baumannii* ST-15 Strains Co-producing OXA-23 and OXA-58 in South America

**DOI:** 10.1007/s00284-026-05160-1

**Published:** 2026-10-06

**Authors:** Aline Madeira Marques Saraiva, Amália Raiana Fonseca Lobato, Emanoele Saraiva Pereira, Maria Paula Cruz Schneider, Diego Assis das Graças, Adonney Allan de Oliveira Veras, Artur Silva, Danielle Murici Brasiliense, Rafael Azevedo Baraúna

**Affiliations:** 1 Biological Engineering Laboratory, Guamá Science and Technology Park, Belém, 66075-750 Brazil; 2 Bacterial Resistance Laboratory, Bacteriology Section, Evandro Chagas Institute, 67030-000 Ananindeua, Brazil; 3 Center for Genomics and Systems Biology, Federal University of Pará, Belém, 66075-110 Brazil

## Abstract

*Acinetobacter baumannii* is a high-priority pathogen due to its global dissemination and antimicrobial resistance. This work describes the first report of *A. baumannii* ST-15 isolates belonging to the International Clone 4 group, that exhibited an MDR profile with the co-production of OXA-23 and OXA-58 enzymes. Strains were isolated from an intensive care unit in a hospital of the Brazilian Amazon. Eight strains were recovered from an intensive care unit, showing high clonal stability across diverse clinical sources, including tracheal aspirates, urine, and blood, which underscores the high versatility and systemic dissemination potential of this lineage.The *bla*_OXA−23_ and *bla*_OXA−58_ genes were detected in all strains. *A. baumannii* isolates AB_OXA58_IEC07 and AB_OXA58_IEC02 were selected for hybrid genome sequencing and plasmid reconstruction, respectively, as representative samples of the outbreak. The reconstructed mobilome revealed a genome size of 4,110,050 bp, a GC content of 39.2%, and 3846 coding sequences for the reference strain, while four distinct plasmids were fully resolved.The *bla*_OXA−58_ gene was detected flanked by IS*Aba*3 and IS6-like elements.*p*dif modules were identified in another plasmid, together with mobilization and replication genes. The *tet39* gene was also identified in a novel plasmid that is likewise mobilizable. VFDB found several genes related to biofilm formation and virulence. The identification of these high-risk genetic determinants within a monoclonal outbreak, involving diverse clinical sites, emphasizes the successful persistence of the ST-15 lineage in the hospital environment. This study describes a rare co-production of oxacillinases, highlighting the importance of constant genomic surveillance in monitoring the emergence of antibiotic-resistant pathogens that could pose a threat on a regional or global scale.

**Supplementary Information:**

The online version contains supplementary material available at https://doi.org/10.1007/s00284-026-05160-1.

## Introduction

*Acinetobacter baumannii* is a high-priority pathogen due to its global dissemination and antimicrobial resistance [1]. It is one of the main causes of healthcare-associated infections [1, 2]. The resistance phenotype is primarily associated with the production of class D beta-lactamases, known as oxacillinases (OXAs), which are encoded by *bla*_OXA_ genes [2]. The. Among acquired oxacillinases in *Acinetobacter baumannii*, *bla*_OXA−23−_like is the most frequently reported worldwide [3], whereas *bla*_OXA−24/40−_like, *bla*_OXA−58−_like, *bla*_OXA−143−_like, and *bla*_OXA−235−_like have been described less frequentlyn in the literature. In addition, *bla*_OXA−ADC_ (*ampC*) and *bla*_OXA−Ab_ (*bla*_OXA−51−_like) are intrinsic to the species [4].

These genes may be carried by Mobile Genetic Elements (MGEs) such as insertion sequences (IS) and transposons. The MGEs can play a key role in regulating the expression of antimicrobial resistance genes (ARGs), including oxacillinases, efflux pump systems, and others [5]. For example, IS*Aba*1 and IS*Aba*125 elements are often associated with over-expression of the *ampC* and *oxaAb* genes [5]. The over-expression of constitutive ARGs poses a problem for clinical treatment, as it typically increases the minimum inhibitory concentration of drugs. Therefore, genomic surveillance is a powerful tool for monitoring the emergence of antibiotic-resistant pathogens, such as *Acinetobacter* spp., that is a potential threat at a regional or global scale [1].

The first broad-spectrum oxacillinase, OXA-23, was identified in a strain isolated in the city of Edinburgh, United Kingdom, in 1985 [6]. Nowadays, *bla*_OXA−23_-like genes are widely distributed and have been reported in several countries including Bulgaria, China, Brazil, Iraq, Afghanistan, France, and others. In 2003, the OXA-58 was identified in a strain isolated in Toulouse, France [6]. In South America, the oxacillinase enzyme was first detected in *Acinetobacter* spp. isolated in Argentina [7]. Current reports, described the co-production of the *bla*_OXA−23_, *bla*_OXA−58_, and *bla*_NDM_ [8], highlighting the emergence of strains that accumulating multiple carbapenem-resistance determinants, including the combination of different classes of oxacillinases and metallo-beta-lactamases within the same isolate [8].

A recent study demonstrated that *A. baumannii* International Clones 5 (IC5) and 7 (IC7) are the most prevalent in South America. This differs from other continents, where IC1 and IC2 are the most commonly reported [3]. The IC5 and IC7 groups are related to several Pasteur scheme sequence types (STs) including the 79, 156, 175, 422, 730, 1163, 1196, for IC5 group and 25, 113, 619, 945, and 1487 for IC7 group. The ST-15, on the other hand, is usually associated with the IC4 group [9]. Although IC5 and IC7 currently dominate the regional prevalence, IC4 (ST-15) remains a significant epidemic lineage in Latin America [10]. In countries like Brazil and Argentina, Clonal Complex 15 (CC15 (IC4)) and CC79 (IC5) continue to be recognized as major endemic vehicles for carbapenemase genes [11], highlighting the persistent clinical relevance of ST-15 despite shifts in clonal dominance [10, 11].

This work presents the first report of *A. baumannii* strains carrying both OXA-23 and OXA-58 enzymes in the Amazon region. They were responsible for an outbreak in a hospital in the Brazilian state of Acre. The strains were highly clonal and exhibited a multidrug resistance (MDR) profile and were classified in the rare ST-15 (IC4).

## Materials and Methods

### Selection of Bacterial Isolates and Antimicrobial Susceptibility Testing

The study included 259 isolates of *Acinetobacter baumannii* received at the Evandro Chagas Institute through the antimicrobial resistance surveillance flow from hospitals in the Northern region (states of Pará and Acre), between 2020 and 2021. After screening for the presence of carbapenemases, eigth isolates co-producing OXA-23 and OXA-58 were further characterized) (Table 1). Isolation was performed from different samples such as tracheal aspirate, urine, and blood. Antimicrobial susceptibility testing was performed using the disc diffusion method according to Clinical and Laboratory Standards Institute (CLSI) guidelines [12]. The following antibiotics were tested: piperacillin (100 µg mL^− 1^), ampicillin-sulbactam (10/10 µg), piperacillin-tazobactam (100/10 µg), ticarcillin-clavulanate (75/10 µg), ceftazidime (30 µg), cefepime (30 µg), cefotaxime (30 µg), ceftriaxone (30 µg), imipenem (10 µg), gentamicin (10 µg), tobramycin (10 µg), amikacin (30 µg), tetracycline (30 µg), and ciprofloxacin (5 µg). *Escherichia coli* ATCC 25922 and *Pseudomonas aeruginosa* ATCC 27853 were used for quality control, and CLSI breakpoints were used to classify strains as susceptible (S), intermediate (I) or resistant (R).

### Screening for β-lactamase Genes

The detection of the *bla*_OXA−23_-like, *bla*_OXA−24_-like, *bla*_OXA−51_-like, *bla*_OXA−58_-like, and *bla*_OXA−143_-like genes was performed using a multiplex Polymerase Chain Reaction (PCR) assay as described by Woodford and colleagues [13]. For the detection of the *bla*_KPC_, *bla*_NDM_, *bla*_IMP_, and *bla*_VIM_ genes, the multiplex PCR protocol developed by Pasteran and colleagues was used [14]. Finally, the detection of the *bla*_GES_ gene was performed using the pair of primers developed by Poirel and colleagues [15]. All reactions were carried out in the VeritiPro Thermal Cycler (Applied Biosystems), using previously positive samples sequenced by Sanger as positive controls for target confirmation.

### Multi-Locus Sequence Typing

Multi-Locus Sequence Typing (MLST) analysis was performed using the Institut Pasteur scheme [16]. Seven primer pairs were used to amplify internal fragments of the *rpoB*, *rplB*, *recA*, *pryG*, *gltA*, *fusA*, and *cpn60* genes. PCR products were purified using the ExoSAP-IT kit (Thermo Fisher Scietific) and sequenced by Sanger sequencing on a ABI 3500 Genetic Analyzer (Thermo Fisher Scietific). The cycling conditions comprised an initial denaturation step at 94 °C for 2 min, followed by 35 cycles of denaturation at 94 °C for 30 s, annealing at 50 °C for 30 s, and extension at 72 °C for 30 s, concluding with a final extension step at 72 °C for 5 min. The reaction was performed in a GeneAmp 9700 PCR system (Applied Biosystems). sequenced as previously described. The DNA sequences were analyzed using BioEdit v.5.0.9 [17]. Consensus sequences were generated from forward and reverse reads whenever available. To resolve potential ambiguities, a stringent trimming protocol was applied, and allelic assignment was based on the comparison of high-quality internal fragments with reference alleles from the PubMLST database [18], allowing determination of the Sequence Type (ST) of each isolate.

A maximum likelihood tree was calculated based on the aforementioned genes using the NGPhylogeny web platform [19]. The alignment was performed using MAFFT Software, Version 7 (Multiple Sequence Alignment) [20] followed by a data curation using BMGE (Block Mapping and Gathering with Entropy) [21]. The tree was calculated using the PhyML software with 1,000 bootstrap replicates and visualized using Newick Display software [22–24]. This Maximum Likelihood (ML) approach was preferred over e-BURST diagrams to ensure higher phylogenetic resolution by analyzing the complete nucleotide sequences of the housekeeping genes, which allows for a more precise evaluation of the genetic proximity among isolates sharing the same ST and confirms the monoclonal origin of the outbreak.

### Whole Genome Sequencing and Bioinformatics

Genome sequencing was performed in *A. baumannii* AB_OXA58_IEC07 on the PromethION 2 Solo platform (Oxford Nanopore Technologies) using the Rapid Sequencing DNA V14 Barcoding kit (96V14), following the manufacture’s instructions. Quality assessment was performed using NanoPlot v.1.46.1 [25] and low-quality bases were removed with Nanofilt v.2.8.0 [26]. The genome assembly was performed using Flye v.2.9.2 [27], with the *nano-raw* and *genome-size* parameters. Genome completeness was evaluated using BUSCO [28] and gene annotation was performed with Prokka v.1.14.6 [29] (Figure S1). For coding sequence (CDS) identification, coordinates were predicted via Prodigal v.2.6.3 [30], while transfer RNA (tRNA) and transfer-messenger RNA (tmRNA) genes were identified by Aragorn v.1.2.38 [31]. The pipeline further integrated HMMER v.3.3 [32] for Hidden Markov Model profiles, alongside Blast + v.2.12.0 [33] and Infernal v.1.1.4 [34] for structural alignments. Proteins were annotated using the --addgenes parameter for gene nomenclature inclusion in the locus tags. The visual systematization of the bioinformatics stages was consolidated in a methodological flowchart (Figure S1), developed with the support of the Gemini (Google) [35] generative artificial intelligence tool, under the author’s direct supervision and provision of technical parameters.

The following web tools and databases were used for genome analysis: ResFinder v.4.7.2 [36], Virulence Factor Database (VFDB) [37], PathogenWatch [38], PlasmidFinder v.2.1 [39], ISFinder [40], MLST [41], CARD Resistance Gene Identifier (RGI) v.4.0.1 [42]. Plasmid typing and mobility analysis were performed with MOB-suite v.3.1.9 [43, 44], (available in Galaxy v.4.2.0, https://usegalaxy.eu) [45]. Manual curation was conducted using BLASTn against the GenBank database [46]. Structural conservation and synteny were analyzed using Artemis v.18.2.1 and DNA Plotter [47], comparing the reconstructed sequences with reference plasmid pAC161 (Accession: MZ573927.1). Insertion Sequences (ISs) were identified using the ISFinder database [40]. To ensure the accuracy of genomic characterization, all bioinformatic hits were filtered using a minimum threshold of 90% identity and 60% coverage. Blast Ring Image Generator (BRIG) [48] was used to draw genome rings.

### Isolation of Plasmid DNA and Sequencing

After whole genome sequencing, the strains *A. baumannii* AB_OXI58_IEC02, AB_OXI58_IEC03 and AB_OXI58_IEC04 were selected for plasmid isolation using the QIAprep Spin Miniprep kit (QIAGEN), following the manufacture’s protocol. The DNA was quantified and sequenced on the PromethION 2 Solo platform (Oxford Nanopore Technologies) using the Rapid Sequencing DNA V14 Native Barcoding kit (96V14), in a multiplexed run according to the manufacturer’s protocol. The raw sequencing reads were subjected to quality filtering and adapter removal to ensure a minimum Phred score of Q20. Due to insufficient read depth for isolates AB_OXI58_IEC03 and AB_OXI58_IEC04, only isolate AB_OXI58_IEC02 was used for high-quality *de novo* genomic reconstruction. Assemblies were performed using SPAdes v. 4.2.0 [49] for short-read analysis and Unicycler v.0.5.1 [50] (both available in Galaxy v.4.2.0, https://usegalaxy.org ) [45]. The hybrid approach integrated Illumina and PromethION long-reads to resolve repetitive regions and achieve complete plasmid circularization.

Antibiotic Resistance Genes (ARGs) were detected using *staramr* v.0.11.0 [51] (available in Galaxy v.4.2.0, https://usegalaxy.org) [45], and the CARD database v.4.0.1 [42]. Due to the known limitations of traditional incompatibility (Inc) group classification for the *Acinetobacter* genus, which often leads to inconclusive results in platforms such as PlasmidFinder and pMLST, plasmid typing and mobility prediction were performed via MOB-suite v.3.1.9 [43, 44] (available in Galaxy v.4.2.0, https://usegalaxy.eu) [45]. Given the mosaic architecture of the recovered elements, manual curation was conducted using BLASTn against the GenBank database [46]. Structural conservation and synteny were analyzed using Unipro UGENE v.53.1 [52] and Artemis v.18.2.1 [47], comparing the reconstructed sequences with reference plasmids, such as pDETAB5 (Accession: CP072528.1). Insertion Sequences (ISs) were identified using the ISFinder database [40]. Only hits with significant E-values and identity were considered for family-level classification, while the specific genetic context of resistance genes was manually curated using Artemis and BLASTn to ensure host-specific accuracy. Synteny was visualized through Dot Plot analysis and manual mapping of *pdif* sites (XerC/XerD) and ISs (Figure S1). A minimum threshold of 90% identity and 60% coverage was applied to all bioinformatic predictions to ensure the reliability of the results.

## Results

### *A. baumannii* Isolates had an MDR Profile Associated with the Co-production of OXA-23 and OXA-58

*A. baumannii* strains isolated in this study were tested for their susceptibility to seven drug classes. Seven of the eight isolates (87.5%) were susceptible to at least one antibiotic. However, all strains were resistant to at least three drug classes, classifying them as multi drug resistant (MDR) [53] (Table S1). Resistance rates of 37.5%, 25%, and 87.5% were observed for penicillin combined with a β-lactamase inhibitor, tetracycline, and aminoglycosides, respectively. Screening via multiplex PCR and Sanger sequencing confirmed the presence of *bla*_OXA−23_, *bla*_OXA−51_, and *bla*_OXA−58_ by amplification of target genes in all eight isolates (Table 1). These findings were further corroborated by whole-genome sequencing (WGS) and targeted plasmid analysis, which provided the structural context for these determinants. While *bla*_OXA−23_ and *bla*_OXA−51_ were localized to the chromosome, the *bla*_OXA−58_ carbapenemase was associated with mobile extrachromosomal elements, as detailed in the following sections.

**Table 1:** Phenotypic and genotypic profiles of eight *Acinetobacter baumannii* isolates carrying *bla*_OXA−23_, *bla*_OXA−51_ and *bla*_OXA−58_ genes

Isolate	Phenotype (Resistance profile)	Chromossomal genotype (QRDR Mutation)s	Plasmes content (genotype)
AB_OXA58_IEC01	PIP, PTZ, TCC, CAZ, FEP, CTX, CRO, IPM, GEN, TOB, CIP: (R) APS, AMI, TET: (S)	Not analyzed	Not analyzed
AB_OXA58_IEC02	PIP, PTZ, TCC, CAZ, FEP, CTX, CRO, IPM, GEN, TOB, CIP: (R) APS (I) AMI, TET: (S)	Not analyzed	**4 Circular Plasmids**: p56612-1(pAC163): Cryptic (*pdif* sites) p56612-2(pAD745): *bla*_OXA−58_ p56612-3(pAB4826): Stability (TA systems) p56612-4(novel): *tet(39)*
AB_OXA58_IEC03	PIP, PTZ, TCC, CAZ, FEP, CTX, CRO, IPM, GEN, TOB, CIP: (R) APS, AMI, TET: (S)	Not analyzed	Not analyzed
AB_OXA58_IEC04	PIP, APS, PTZ, TCC, CAZ, FEP, CTX, CRO, IPM, GEN, TOB, CIP: (R) AMI (S), TET (S)	Not analyzed	Not analyzed
AB_OXA58_IEC05	PIP, PTZ, TCC, CAZ, FEP, CTX, CRO, IPM, GEN, TOB, CIP: (R) APS, AMI, TET: (S)	Not analyzed	Not analyzed
AB_OXA58_IEC06	PIP, PTZ, TCC, CAZ, FEP, CTX, CRO, IPM, GEN, TOB, CIP: (R) APS, AMI, TET: (S)	Not analyzed	Not analyzed
AB_OXA58_IEC07	PIP, APS, PTZ, TCC, CAZ, FEP, CTX, CRO, IPM, GEN, TOB, AMI, TET, CIP: (R)	*GyrA* (S81L) *ParC* (S84L, V104I, D105E)	**Plasmid Clusters (Fragmented)**: pAB114: none pIEC57529 (pAC161-like): *aac(3)*,* bla*_TEM−1_, *mphE*, *msrE*
AB_OXA58_IEC08	PIP, PTZ, TCC, CAZ, FEP, CTX, CRO, IPM, GEN, TOB, CIP, TET: (R) APS, AMI: (S)	Not analyzed	Not analyzed

R: Resistant; S: Susceptible; I: Intermediate; Piperacilin: PIP; Ampicilin/ Sulbactam: APS; Piperacilin/Tazobactam: PIP/TAZ; Ticarcillin/ Clavulanate: TCC; Ceftazidime: CAZ; Cefepime: FEP; Cefotaxime: CTX; Ceftriaxone: CRO; Imipenem: IPM; Gentamicin: GEN; Tobramicin: TOB; Amikacin: AMI; Tetraciclin: TET; Ciprofloxacin: CIP; QRDR: Quinolone Resistance-Determining Region

Note: Three strains were susceptible to ampicillin+sulbactam (AB_OXA58_IEC03, AB_OXA58_IEC05, and AB_OXA58_IEC06), one strain was susceptible to amicacin (AB_OXA58_IEC08), and six strains were susceptible to tetracycline (AB_OXA58_IEC01, AB_OXA58_IEC02, AB_OXA58_IEC03, AB_OXA58_IEC04, AB_OXA58_IEC05 and AB_OXA58_IEC06)

Not analyzed: Genomic analysis (WGS or plasmid-seq) was performed only for representative isolates AB_OXA58_IEC07 and AB_OXA58_IEC02. The presence of *bla*_OXA−23_, *bla*_OXA−51_ and *bla*_OXA−58_ was confirmed in all eight isolates via multiplex PCR

Phenotype: Resistance profile obtained through VITEK-2 and disk diffusion tests

Genotype: Chromosomal determinants (QRDR mutations and *bla*_OXA−23_) were identified in all isolates

Plasmidial Architecture: For isolate AB_OXA58_IEC02, the extrachromosomal content was resolved into four circular plasmids (p AB_OXA58_IEC02_1 to 4) using hybrid assembly (Illumina/PromethION). For AB_OXA58_IEC07 isolate, plasmid content reflects clusters identified via MOB-suite from WGS data

### The bacterial Outbreak was Caused by Highly Clonal Strains

All *A. baumannii* strains were classified as ST-15 according to PubMLST [18]. Initial automated analysis of the *recA* and *rplB* loci suggested the presence of alternative alleles, such as alleles 8 and 617 for *recA*, and allele 53 for *rplB*. However, subsequent manual curation and alignment of the consensus sequences revealed that these assignments were bioinformatic artifacts. Within the high-quality sequenced regions, all isolates exhibited 100% identity (zero mismatches) with the standard alleles for the ST-15 profile (*recA* allele 3 and *rplB* allele 5). These discrepancies were attributed to the partial nature of the Sanger sequences, which shared identical conserved internal fragments with the automatically assigned profiles (Table 2).

**Fig. 1:**
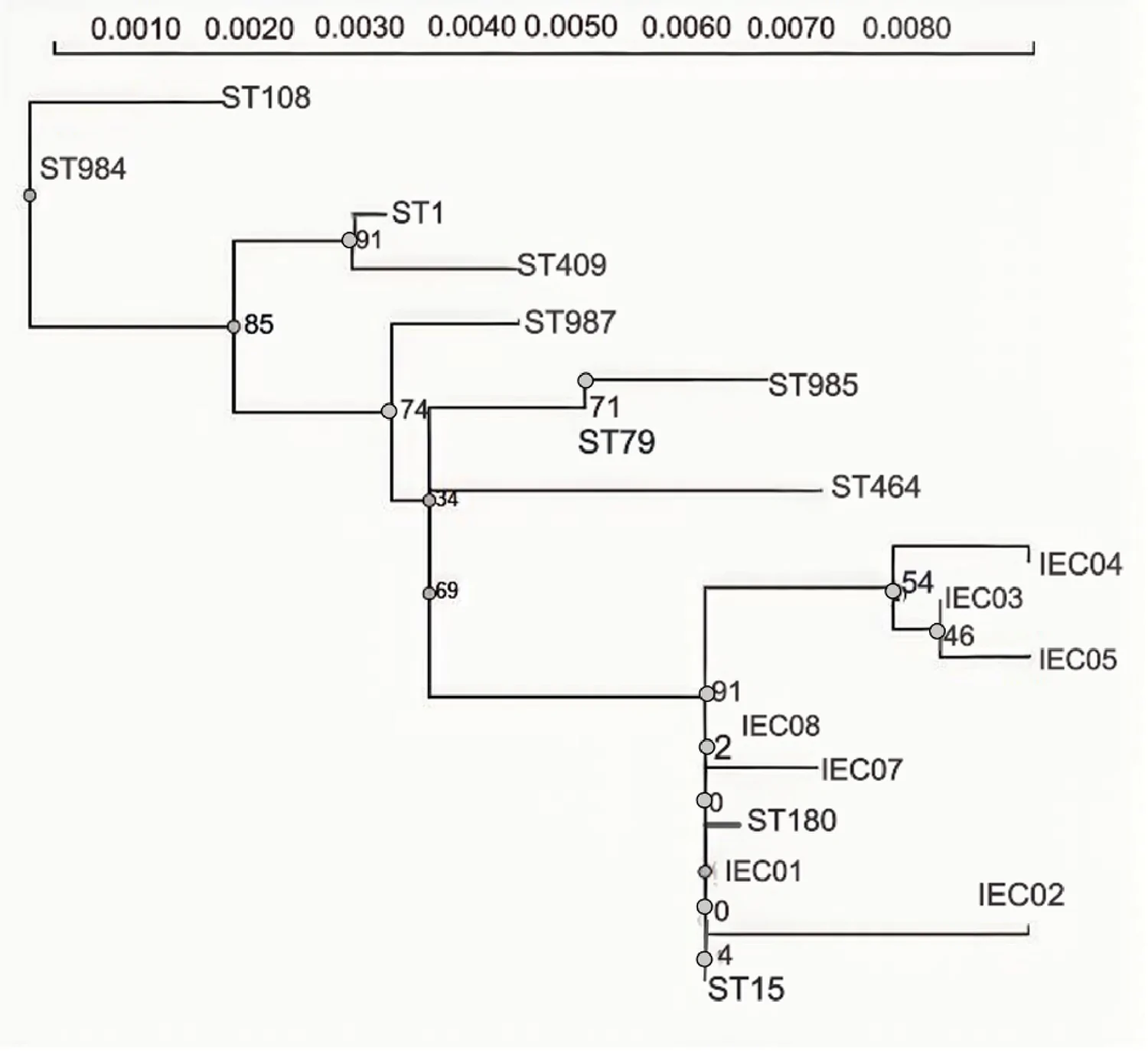
Maximum likelihood tree comparing seven isolates obtained in this study with representative sequences of ST-1, ST-15, ST-79, ST-108, ST-180, ST-409, ST-464, ST-984, ST-985, and ST-987. The sequences are from the *rpoB*,* rplB*,* recA*,* pyrG*,* gltA*,* fusA*, and *cpn60* genes. Bootstrap values above 500 are shown in the phylogenetic tree. All seven *A. baumannii* strains were found to be part of a clade with the ST-15 and ST-180 representative sequences, with a bootstrap value of 91%. AB_OXA58_IEC(0x) is listed in the tree under the designation IEC(0x)

The maximum likelihood tree clustered the samples together with reference isolates of the ST-15 and ST-180 (Fig. 1). The only difference between these two STs is the *recA* gene. Allele 3 is found in ST-15, while allele 30 is found in ST-180. Therefore, the phylogenetic tree confirms the ST classification of the isolates. Isolate AB_OXA58_IEC 06 was not included in the phylogenetic analysis because, despite our efforts, we were unable to amplify and sequence the *gtlA* gene.

**Table 2:** Description of the allelic profile of the *A. baumannii* strains isolated in this study. Despite our efforts, we were unable to sequence the *gltA* gene of strain AB_OXA58_IEC06. The allelic profile of all other samples was consistent with ST-15

Sample ID	Site of isolation	Allelic profile							ST
		rpoB	rplB	recA	pyrG	gltA	fusA	cpn60	
**IEC01**	Tracheal Aspirate	4	5	3	2	8	6	6	15
**IEC02**	Tracheal Aspirate	4	5	3	2	8	6	6	15
**IEC03**	Tracheal Aspirate	4	5	3	2	8	6	6	15
**IEC04**	Tracheal Aspirate	4	5	3	2	8	6	6	15
**IEC05**	Tracheal Aspirate	4	5	3	2	8	6	6	15
**IEC06**	Urine	4	5	3	2	x	6	6	x
**IEC07**	Blood	4	5	3	2	8	6	6	15
**IEC08**	Tracheal Aspirate	4	5	3	2	8	6	6	15

ST: Sequence Type; (*). AB_OXA58_IEC(0x) is listed in the table under the designation IEC(0x). Allelic identification was refined through manual curation and consensus sequence assembly using BioEdit v7.2.5. For isolates where bidirectional sequencing (forward and reverse) was partially available or showed low-confidence ends, allelic assignment was based on high-fidelity internal fragments. All curated sequences exhibited 100% identity (zero mismatches) with the reference alleles in the PubMLST database. Initial bioinformatic artifacts (e.g., automated assignment of alleles 8 or 617 for *recA* and allele 53 for *rplB*) were resolved by confirming identical conserved regions within the CC15 (Clonal Complex 15) lineage

### *A. baumannii* AB_OXA58_IEC07 Carries Several Virulence and Antimicrobial Resistance Genes

**Fig. 2:**
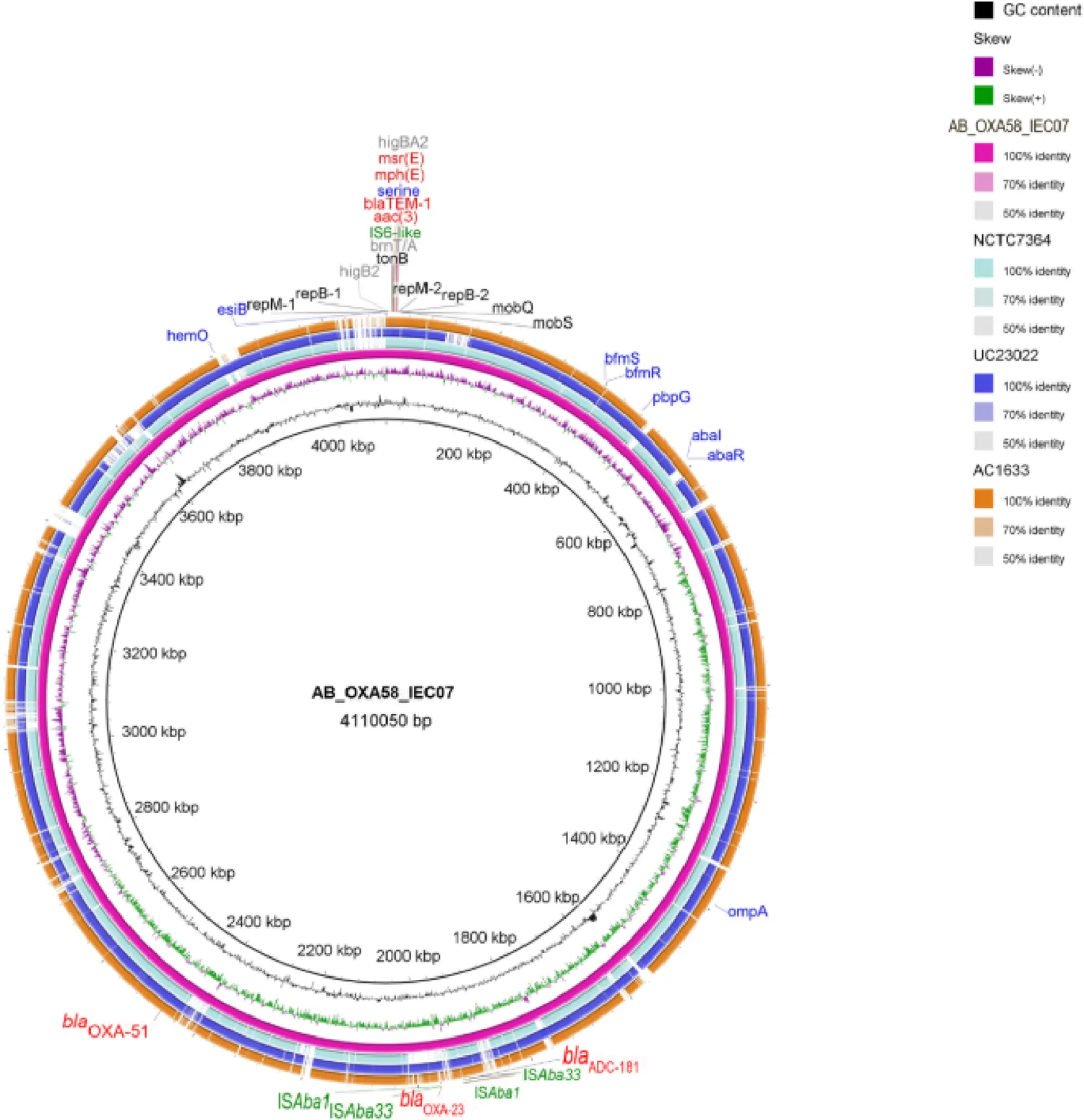
Genomic ring comparing *Acinetobacter baumannii* AB_OXA58_IEC07 with the globally recognized epidemiological reference *A. baumannii* strains NCTC 7364, UC 23,022 and AC 1633 2. The gaps in the rings indicate regions of low similarity to the *A. baumannii* AB_OXA58_IEC07 genome. Antibiotic resistance genes such as *bla*_OXA−23_, *bla*_OXA−51_, and *bla*_ADC−181_ are highlighted in red, while virulence genes such as *ompA*,* hemO*, and *pbpG* are highlighted in blue. Replication and mobilization genes presented in the plasmid AB_OXA58_IEC07_1 are highlited in black and genes related to the Toxin-Antoxin sytems in grayThe insertion sequences associated with the antibiotic resistance genes are highlighted in green. The size of the *A. baumannii* AB_OXA58_IEC07 genome is 4,110,050 bp

**Fig. 3:**
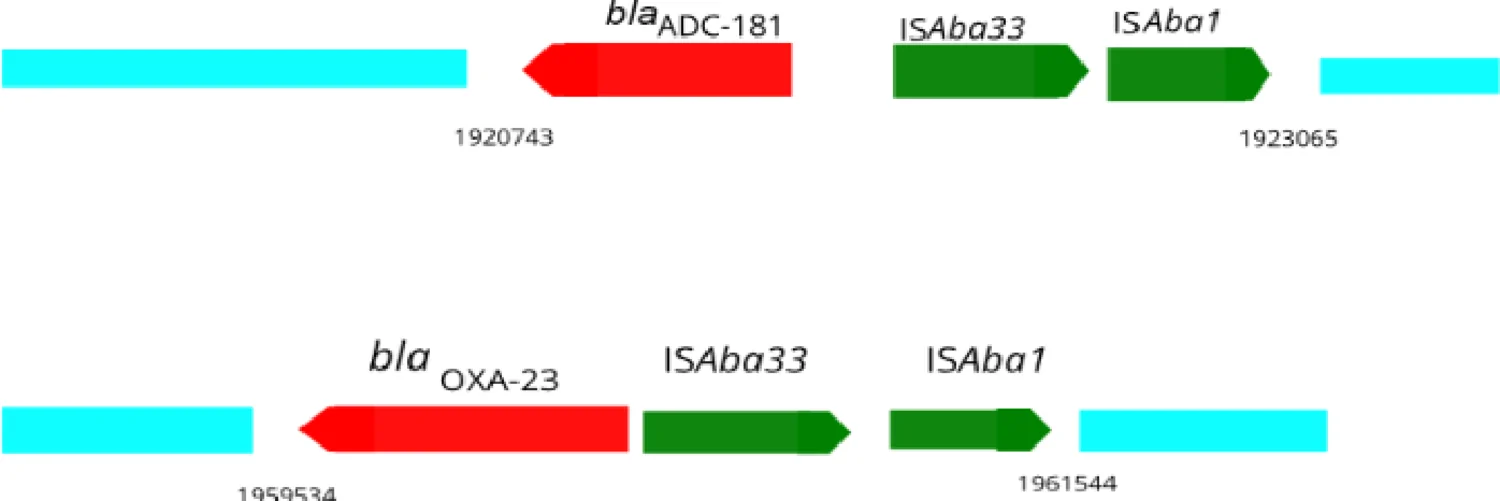
Synteny analysis of antibiotic resistance genes in *A. baumannii* AB_OXA58_IEC07 genome. The *bla*_ADC−181_ and *bla*_OXA−23_ were associated with the IS*Aba*33 and IS*Aba*1 elements. Due to their synteny, it is likely that the promoter of IS*Aba*33 is responsible for regulating the expression of both antibiotic resistance genes. This differs from what has been described in the scientific literature, where IS*Aba*1 is usually associated with this regulation. Further analysis is required

**Fig. 4:**
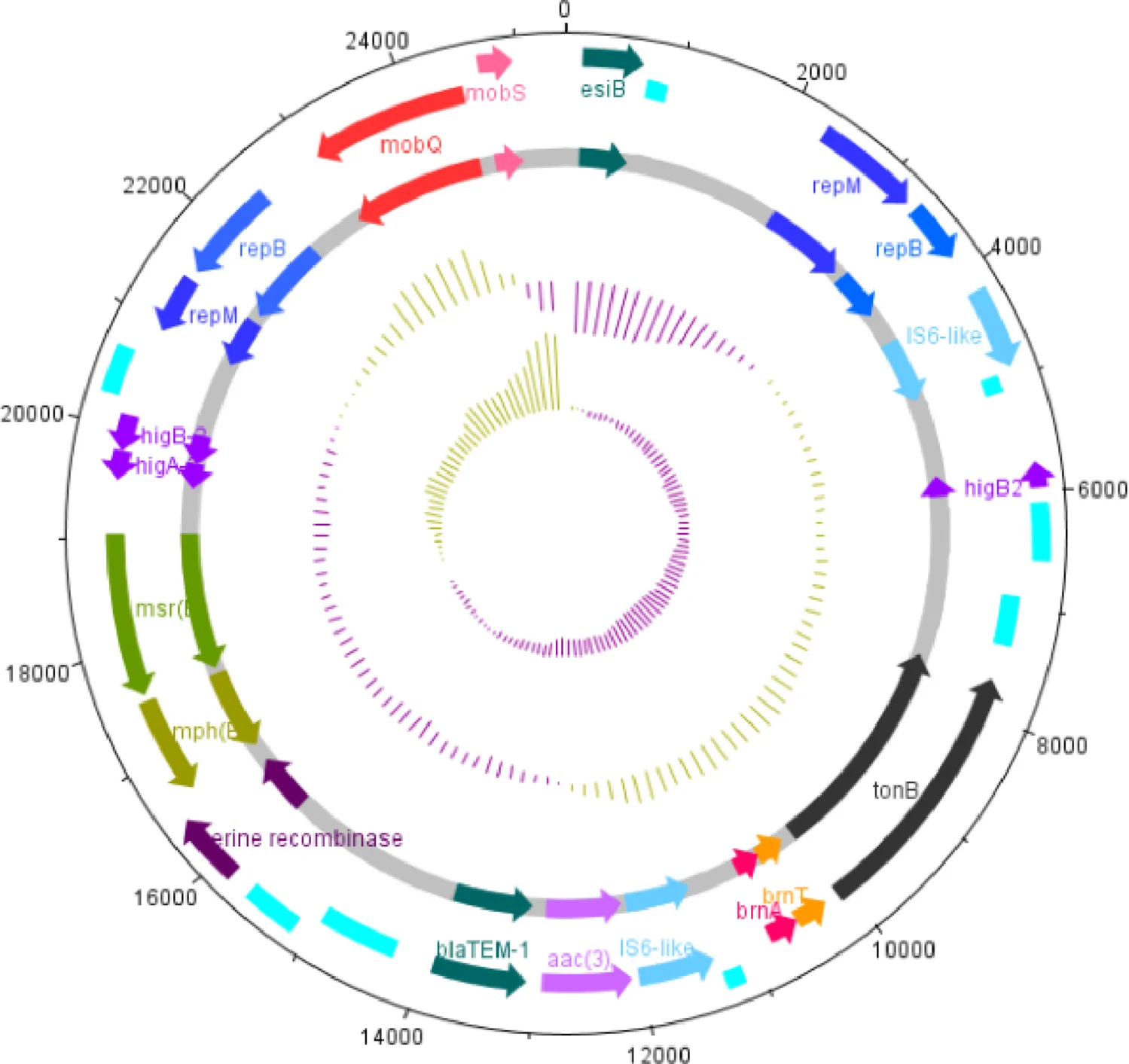
Circular map and genomic organization of plasmid pAB_OXA58_IEC07_1 (pAC161-like). The map illustrates the results of the manual curation of contig_8, reconstructed from the genomic data of *A. baumannii* isolate AB_OXA58_IEC07. The plasmid exhibits a multireplicon structure, characterized by dual replication modules (RepM-RepB) in inverted orientations, ensuring high replication stability. A central resistance cluster is highlighted, harboring *bla*_TEM−1_, aac(3), and the macrolide resistance genes *mph(E)* and *msr(E)*. The presence of curated Type II Toxin-Antitoxin systems (*higBA-2* and *brnAT*) explains the robust maintenance of this replicon within the clinical isolate. The MobQ/MobS mobilization module, resolved through manual analysis of previously ambiguous annotations (e.g., *esiB*), confirms the potential for horizontal gene transfer. The grey inner ring represents the reference plasmid pAC161 (MZ573927.1) used for synteny and structural conservation analysis, and the outer ring represents contig_8 of *A. baumannii* isolate AB_OXA58_IEC07

Due to the high clonality, only one strain (AB_OXA58_IEC07) was selected for genome sequencing. *A. baumannii* AB_OXA58_IEC07 presented a genome with 4,110,050 bp, GC content of 39.2%, and 3,846 coding sequences. Detailed assembly statistics and functional features are summarized in Table 3. According to the PathogenWatch server, it was classified as ST-15. ResFinder detected several resistance genes, including the chromosomally encoded *bla*_OXA−51_, *bla*_ADC−25_, and *bla*_OXA−23_. BLASTn analysis of the *bla*_ADC−25 −_related sequence confirmed the presence of *bla*_ADC−181_ with 100% identity, a gene associated with sequence type ST-15 [11] (Figs. 2 and 3). A total of 21 insertion sequences (IS) were detected by ISFinder (Table S2). They belonged to the IS4, IS5, IS6, IS30, IS66, and IS256 families, and had all previously been described in *A. baumannii* isolates. Virulence factors were identified using VFDB, revealing a repertoire of genes primarily associated with thebiofilm formation, and cellular persistence (Fig. 2). Regarding the resistome, the genomic analysis of isolate AB_OXA58_IEC07 via the CARD platform identified key chromosomal mutations in the Quinolone Resistance-Determining Region (QRDR), specifically the S81L substitution in *gyrA* and S84L in *parC* (along with V104I and D105E) (Table 1). In addition to these mutations, a diverse set of chromosomally encoded resistance genes was characterized, as detailed in Table 4. Furthermore, plasmid typing based on WGS data characterized two distinct extrachromosomal elements. The first, pAB114 (*rep_cluster_718*) was identified as a non-mobilizable, incomplete plasmid of 32,6 kb (located in contig_6). The second, pAC161 (*rep_cluster_1226* and *1282*; secundary cluster id AL237) was classified as a mobilizable, incomplete plasmid of 25,4 kb (located in contig_8), harboring a MOB_Q_-type relaxase. Notably, despite the presence of these scaffolds, the *bla*_OXA−58_ gene was not recovered within these specific WGS-reconstructed plasmids. These preliminary findings are summarized in Table 1 and Table S3 and Fig. 4.

**Table 3:** Genomic assembly statistics and annotation features of *Acinetobacter baumannii* isolate AB_OXA58_IEC07

Genomic Parameter	Value
Sequencing platform	Oxford Nanopore (PromethION 2 Solo)
Assembly software	Flye v.2.9.2
Total bases sequenced	817.3 Mb
Average coverage	204.0x
Genome size (bp)	4,110,050
Number of contigs	3
N50 (bp)	4,052,017
L50	1
G + C content (%)	39.2%
Total genes	3,933
Coding sequences (CDS)	3,846
rRNAs / tRNAs / tmRNA	12 / 74 / 1

**Table 4:** Chromossomal antibiotic resistance and virulence-associated genes indetified in *Acinetobacter baumannii* AB_OXA58_IEC07

GENOMIC LOCATION	CATEGORY	GENOTYPE	PHENOTYPIC CHARACTERIZATION / BIOLOGICAL ROLE
AB_OXA58_IEC07 CHROMOSOME	Beta-lactam Resistance	*bla*_ADC−181_, *bla*_OXA−23_, *bla*_OXA−51,_*bla*_TEM−1_	Antibiotic inactivation
	Aminoglycoside Resistance	*armA*	High-level multidrug resistance (16 S rRNA methyltransferase)
	Resistance-nodulation-cell division (RND)	*adeIJK*,* adeF*,* adeN*,* adeR*	Antibiotic efflux
	small multidrug resistance (SMR)	*abeS*,	Antibiotic efflux
	Major facilitator superfamily (MFS)	*Acinetobacter baumannii* AbaF, *Acinetobacter baumannii* AbaQ, *Acinetobacter baumannii* AmvA	Antibiotic efflux
	Virulence (Adhesion/ Biofilm)	*ompA*, *pbpG*, *bfp*, *pgaA*, *pgaB*, *pgaC*, *pgaD*	Biofilm formation, cell adhesion, and environmental persistence
	Virulence (Iron Acquisition)	*hemO*, *basA*, *basB*, *basC*, *basD*, *basF*, *basG*, *basH*, *basI*, *basJ*, *bauA*, *bauB*, *bauC*, *bauD*, *bauE*, *bauF*	Siderophore-mediated iron uptake (Acinetobactin)
	Virulence (Secretion/ Capsule)	*vgrG* (T6SS), *hcp* (T6SS), *cps* (Capsule)	Type VI Secretion System (T6SS) and immune system evasion

The insertion sequences IS*Aba1*, IS*Aba33* were detected in the chromosome of *A. baumannii* AB_OXA58_IEC07 and may contribute to the mobilization and ehanced expression of chromosomal resistance determinants, especially β-lactamase genes

**Fig. 5:**
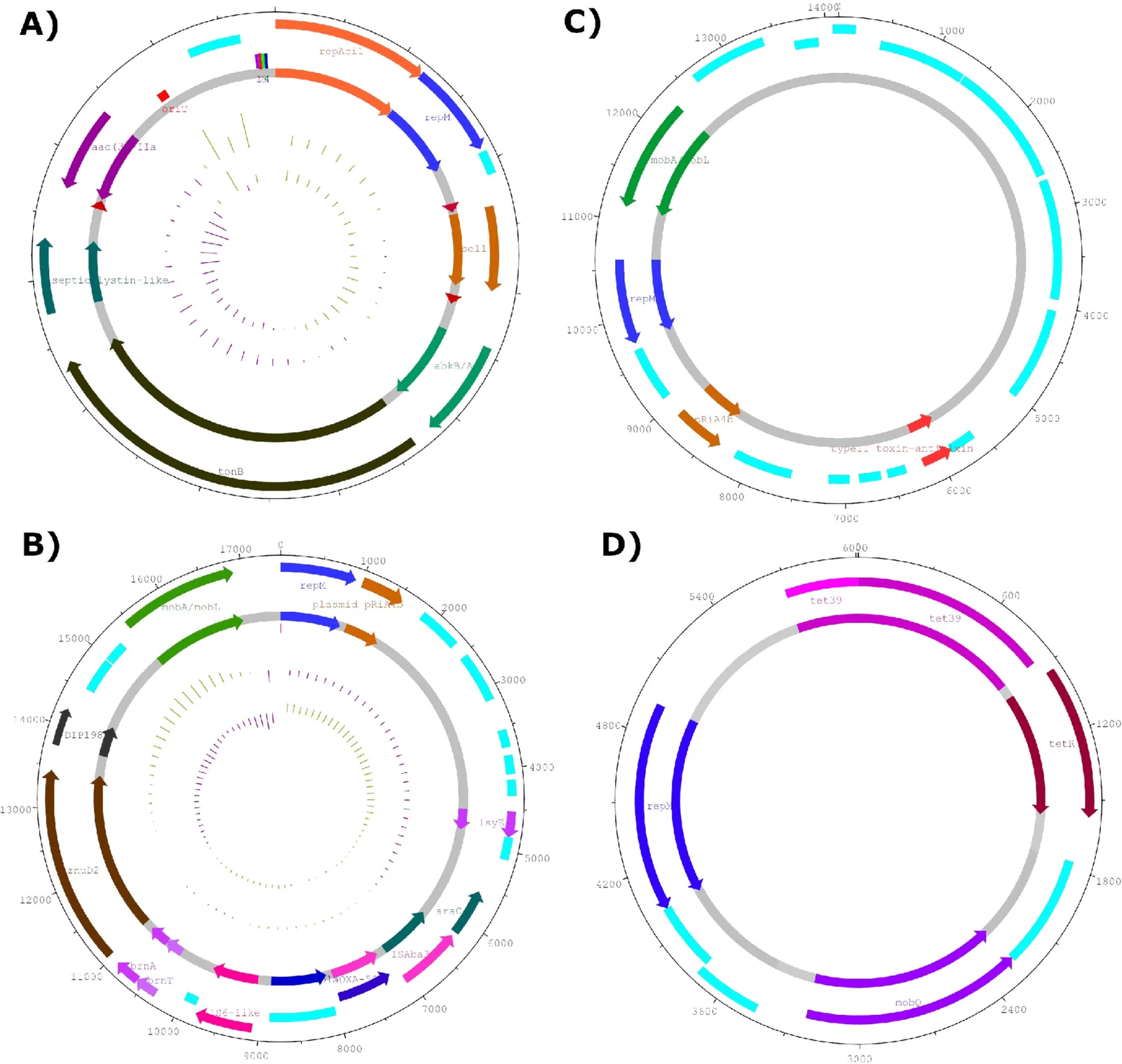
Structural synteny and genomic characterization of plasmids reconstructed from *A. baumannii* isolate AB_OXA58_IEC02. (A) Structural map of the pAB_OXA58_IEC02_1 plasmid. (**A**) Circular representation of the pAB_OXA58_IEC02_1 replicon (~ 2,68 kb). The inner ring displays identified ORFs from isolate AB_OXA58_IEC02, with arrows indicating the direction of transcription: replication genes (orange/blue), stability systems (teal), virulence factors (dark brown/dark green), and resistance determinants (purple). Red triangles highlight the position of XerC/D-like (*pdif*) recombination sites flanking modular segments such as *sel1* and *aac (3) - IIa*. The outer ring represents the reference plasmid pWM98-1 (CP059301.1), used as a scaffold for synteny validation and manual curation in Artemis. Internal tracks represent GC content (black) and GC skew (purple/olive). (**B**) Plasmid pAB_OXA58_IEC02_2: The map details the genetic environment of the *bla*_OXA−58_ gene (blue) within a ~ 1.75 kb plasmid. Note the presence of IS*Aba3* and IS6-like elements (pink arrows) flanking the resistance determinant, characterizing a specialized mobilization unit, mobA/L (green). The backbone features a multireplicon structure (*repM* and *pRiA4b*), the *brnAT* toxin-antitoxin system for plasmid stability, and a TonB-dependent receptor (*znuD2*). The outer ring represents the reference plasmid p2UC20804 (CP076809.1). (**C**) Plasmid p pAB_OXA58_IEC02_3: Circular representation of a ~ 1.7 kb cryptic plasmid. The structural analysis reveals a multireplicon backbone comprising *repM* and *pRiA4b* modules (blue/brown). Stability is maintained by a Type II Toxin-Antitoxin system (red arrows), and potential horizontal transfer is facilitated by the *mobA/mobL* mobilization module (green arrows). The outer ring represents the reference plasmid pAB021-A001-1(CP136184.1). (**D**) Plasmid pAB_OXA58_IEC02_4 (novel): A ~ 1,4 kb small replicon dedicated to tetracycline resistance via the *tet(39)* gene (pink arrows) and its regulator *tetR* (red arrows). The backbone consists of a RepM replication protein (blue arrows) and a Mob_Q_-type relaxase, *mobA/mobL* mobilization module (purple arrows). The outer ring represents the reference plasmid pAB021-A049-4 (CP136177.1), used as a scaffold with 90,07% of identity

The target plasmid isolation and sequencing of *A. baumannii* AB_OXA58_IEC02 successfully resolved the initial discrepancy between PCR detection and the draft genome assembly (Figure S3). Using a hybrid assembly approach (Unicycler), the extrachromosomal content was elucidated into four distinct circular plasmids, providing a more precise characterization than the initial fragmented short-read assembly (features are summarized in Tabel 1 and are detailed in Figure S2, Table S4 and Fig. 5).

The primary resistance determinant, *bla*_OXA−58_, was identified within a 1.749 bp mobilizable plasmid (pAB_OXA58_IEC02_2, cluster AD745), flanked by a single IS*Aba3* (IS4 family) and an IS6-like element. Manual curation and ISFinder analysis confirmed that this mosaic architecture exhibits 100% identity with reference p2UC20804 (CP072528.1) (Fig. 5B). This refined structure, resolved only through hybrid sequencing, replaces the initial 89.8 kb fragmented scaffold (cluster AC861) – identified on the analysis of SPAdes assembly approach whit MOB-recon, and detailed in Figure S2.

Additionally, a 2.686 bp mobilizable plasmid was identified (pAB_OXA58_IEC02_1, cluster AC163). Synteny analysis with the pWM98-1 (CP116389) reference revealed a complex mosaic architecture containing XerC/XerD *pdif* recombination sites and a cluster of four integrons (Fig. 5A). Two other small elements were characterized: a 1.738 bp plasmid (pAB_OXA58_IEC02_3, cluster AB826) harboring stability modules (toxin-antitoxin systems) and *mobA/L* genes (Fig. 5C), and a novel 1.440 bp mobilizable plasmid (pAB_OXA58_IEC02_4) carrying the tetracycline resistance gene *tet (39)* and a *mobQ* module (Fig. 5D).

These findings highlight a specialized and plastic plasmid population, where the coexistence of multiple small mobilizable elements, anchored by *pdif* sites and IS elements, drives the multidrug-resistant (MDR) phenotype of the studied ST-15 clone.

## Discussion

*A. baumannii* is a critical opportunistic pathogen, primarily relatedwith healthcare-associated infections (HAI) in immunocompromised or critically ill patients [1]. Treatment can be challenging due to the emergence of MDR, XDR and pan drug resistant (PDR) isolates [54, 55]. The species is listed in the ESKAPE group of priority pathogens and was responsible for 1.27 million deaths worldwide in 2019 [56]. In Brazil, the epidemiological scenario is particularly alarming; while studiesin São Paulo reported60.7% of isolates as XDR [57], data from Goiânia showed a staggering 91.1% MDR prevalence [58]. These regional disparities underscore the high selective pressure within Brazilian hospitals, leading to the widespread circulation of high-risk clones [57, 58].

Our findings align with this critical trend, phylogenetic analysis and molecular typingbased on the Pasteur scheme confirmed the prevalence of Clonal Complex 4 (CC4), specifically identified as ST-15 and its single-locus variant (SLV), ST-180 (Fig. 1). This close genetic relationship aligns with Brazilian molecular epidemiology, where CC4 lineages (including ST-15, ST-180, and ST-318) are frequently linked to the dissemination of carbapenemases and aminoglycoside resistance genes [59]. This is consistent with previous studies that identified the ST-109, ST-162, ST-15, and ST-318 as the most prevalent in South America [60]. The identification of ST-15 across diverse clinical sources, including tracheal aspirates, urine, and blood, underscores the high versatility and systemic dissemination potential of thi lineage. This clonal stability suggests that a single, well-adapted population is circulating within the hospital environment, capable of colonizing and infecting multiple anatomical sites, which poses a significant challenge for infection control measures.

The isolates exhibited a robust MDR profile, characterized by total resistance to fluoroquinolones (0 mm inhibition zone – Table S1) and decreased susceptibility to carbapenems. This severe resistance phenotype is directly supported by the synergistic action of the chromosomal mutations identified in the QRDR (GyrA S81L and ParC S84L – Table 1) and the presence of the AdeIJK efflux system (Table 4) [61] found in the isolate AB_OXA58_IEC07. Notably, the identification of the *adeN* repressor—a key regulator of the only RND pump in *A. baumannii* with significant constitutive expression. (Table 4) [61] —suggests a stable, intrinsic contribution to the MDR profile. Unlike inducible systems, the AdeN-regulated AdeIJK pump provides a baseline extrusion level that effectively reduces intracellular drug concentrations, thereby enhancing the protective effect of the modified topoisomerases targets [61].

This chromosomal strategy is further amplified by a potent enzymatic repertoire. While carbapenem resistance in South American clinical is typically driven by *bla*_OXA−23_ and sometimes *bla*_OXA−58_ [62], our ST-15 isolates exhibit exhibit a rare and potentially synergistic co-production *bla*_OXA−23_*- bla*_OXA−58_. Genomic analysis revealed that the *bla*_OXA−23_ (annotated as a *bla*_OXA−23_-like/*bla*_OXA−133_ variant) is localized within a complex tandem arrangement of IS elements. We identified an IS*Aba33* element (positions 1960454–1961023) localized immediately upstream of the carbapenemase gene (positions 1959534–1960412). Interestingly, this IS*Aba33* element is associated with IS*Aba1* (positions 1961110–1961544), sharing 88% identity with it according to the ISFinder database and both are carried by the Tn2008 transposon (Figs. 2 and 3) [63].

Althought IS*Aba*1 is primarily found in *Acinetobacter* spp., and has been widely reported to regulate gene expression via outward-directed promoters [64], in our strain the close proximity of IS*Aba33* (approximately 42 bp uppstream of the *bla*_OXA−23_ start codon) strongly suggest that IS*Aba33* is the primary driver of gene overexpression in this isolate. This unique synteny, potentially representing an IS*Aba33* insertion within or adjacent to an IS*Aba1*-related structure, highlights a high-level promoter redundancy that likely ensures the robust carbapenem-resistant phenotype of the ST-15 lineage. Interestingly, an identical synteny was observed for the cephalosporinase *bla*_ADC−181_ (positions 1920743–1921894), which is also flanked by the IS*Aba1*-IS*Aba33* tandem. The conservation of this hybrid promoter module in different genomic loci suggests it is a stable and potent mobile unit. To te best of our knowledge, this is the first report of this gene synteny in *Acinetobacter* spp (Fig. 3). In contrast, the intrinsic *bla*_OXA−51_ gene (positions 2463257–2464081) lacked any adjacent IS elements; the nearest insertion sequence, IS*Aba42*, was located approximately 88 kb away, precluding any exogenous promoter influence. These findings confirm that while *bla*_OXA−51_ provides only basal resistance, the high-level resistance observed is a direct result of the IS-mediated overexpression of *bla*_OXA−23_ and *bla*_ADC−181_, further augmented by the plasmid-borne *bla*_OXA−58_.

Complementing the chromosomal MDR core, the resistome of isolate AB_OXA58_IEC07 is further expanded by the presence of a robust, mobilizable replicon, pAB_OXA58_IEC07_1 (pAC161-like). Recovered from contig_8, this ~ 25.4 kb plasmid acts as a resistance hub, harboring a diverse array of enzymatic determinants. Genomic annotation revealed that this plasmid carries the broad-spectrum beta-lactamase *bla*_TEM−1_ and the aminoglycoside modifier *aac (3)*, alongside a specialized macrolide resistance cluster comprising *mph(E)* and *msr(E)*. This plasmid exhibits a multireplicon system and a MOB_Q_-type relaxase. The organization composed of two initiation and control modules (*repM* and *repB*) belonging to the RepAci4 family, combined with the presence of Type II Toxin-Antitoxin (TA) systems, related with stress response, virulence, maintenance and persistence of the plasmisds [65]. The presence of the genes TA *higBA-2* - most prevalente in *A. baumannii* - and *brnAT*, suggests a robust post-segregational killing mechanism [66] (Table S3 and Fig. 4).

Beyond stability, the plasmid carries the virulence factor *esiB*, which possesses SEL1 (Suppressor of lin-12-like) domain involved in host immune system evasion [67], and the *tonB* receptor, iron acquisition in nutrient-restricted environments, such as human tissue during infection [68]. The integration of these adaptation, antibiotic and virulence genes into a highly stable and potentially mobilizable replicon (via the *mobQS* module) reinforces the evolutionary success of the ST-15 lineage.

The genomic architecture of isolates suggests a dynamic process of gene acquisition and maintenance, which is highly prevalent in South American belonguing to Homology Groups 4 (GR4) [65]. Our hybrid assembly approach revealed that the mobilome of isolate AB_OXA58_IEC02 is composed of four small circular plasmids, rather than a single large scaffold as initially suggested by short-read data (Figure S2). Among these, the p AB_OXA58_IEC02_2(1.7 kb) stands out for carrying the *bla*_OXA−58_ gene. Despite its reduced size, this mobilizable replicon harbors a functional MOB_Q_-type relaxase and exhibiting a mosaic architecture, flanked by IS*Aba3* and IS6-like elements (Fig. 5B). Manual curation and sequence alignment performed in UGENE v.4.1.0 [52] confirmed that this specific module shares 100% identity with the reference p2UC20804 (P076809.1) and conserved regions of pDETAB5 (BioSample ID: SAMN18498586).

The mobilization potential is further evidenced by the presence of XerC/D-like (*pdif*) recombination sites. These sequences flank discrete DNA segments know as *dif-*modules, which are recognized by host enzymes that catalyze site-specific integration and excision events [69, 70]. This modular architecture is particularly evident in pAB_OXA58_IEC02_1, where *pdif* sites flank *sel1* replication gene - located within a smaller, high-confidence contig that is physically integrated into the larger plasmid structure - within RelE/ParE an *abkA/abkB* systems family toxin-antitoxin (TA), caracterized by aroud 86% of the plasmids of the GR4 [65]. The co-localization of the TonB-dependent receptor and septicolysin-like genes with the *aac (3) - IIa* determinant highlights a functional clustering of virulence and resistance determinants. This arrangement ensures that metabolic advantages (iron uptake) and defensive weapons (toxin-antitoxin and carbapenemase/aminoglycoside resistance) are co-selected and efficiently disseminated and may confer stability and succes of the isolates [65] (Fig. 5A).

These recombinase-associated sites, facilitate the movement of DNA modules carrying ARGs. The identification of a *tet39* localized at distinct 1.4 kb mobilizable plasmid (pAB_OXA58_IEC02_4), and the presence of a MOB_Q_-type relaxase (Fig. 5D) aligns with the mobilization of tetracycline resistance-related gene *tet39* [69] and the framework described for South American *A. baumannii* plasmids [65]. And in Figuure 5 C, the genetic arragement suggest the presence of mmobile elements associated with plasmid dissemmination. This complex extrachromosomal landscape, characterized by multiple small plasmids rather than a single MDR element, likely provides a fitness advantage, by reducing the metabolic burden while allowing for rapid genomic rearrangements in response to antibiotic pressure [71]. This modularity facilitates the independent loss or acquisition of specific resistance determinants, optimizing bacterial survival under fluctuating clinical stressors [70].

In addition to the capacity for horizontal dissemination of resitance determinants, persistence in the clinical niche is further enhanced by a Type VI Secretion System (T6SS), a contact-dependent antibacterial system that delivers toxic effectors into neighboring cells and promoters competitive advantage [72, 73]. In *A. baumannii*, the T6SS is often regulated Quorum Sensing (QS), mediated by the two-component system AbaI/AbaR. The enzyme AbaI catalyzes the production of N-acyl-homoserine lactone (AHL), which upon reaching a concentration threshold, binds to the AbaR receptor to activate a cascade of reactions that modutation motility and T6SS activity [72, 73].

Complementing this offensive strategy, our isolates possess a robust defensive repertoire. We confirmed the presence of a complete and functional *ompA* gene. As the most abundant outer membrane protein in *A. baumannii*, OmpA plays a dual role in structural integrity and clinical pathogenesis, influencing adhesion to surfaces, serum resistance, and invasion of host cells [74]. Furthermore, OmpA functions as a transmembrane porin; its downregulation is a known mechanism for modifying membrane permeability and reducing antimicrobial adsorption [75]. Alongside OmpA, the *csuE* gene—essential for the pili system and biofilm formation [76] — and the production of extracellular polysaccharides (EPS) provide a defensive shield against external attacks and clinical stress [72].

Finally, iron acquisition was identified as another virulence mechanism. *A. baumannii* AB_OXA58_IEC07 carries a heme oxygenase (*hemO*) that imports and degrades heme subunits to release iron for bacterial growth [77]. This ‘mobilize-and-compete’ strategy, combining a mobilizable platform (MOB_Q_) with aggressive weapons (T6SS) and defensive barriers (biofilm/OmpA), could explains the evolutionary success and persistence of the isolates.

## Conclusion

The indiscriminate use of antimicrobials has caused an unprecedented public health crisis. *Acinetobacter baumannii* is one of the most relevant opportunistic pathogens associated with health care-associated infections, frequently exhibiting carbapenemase resistance. In this study, we reported the first MDR *A. baumannii* ST-15 strains co-producing OXA-23 and OXA-58 in the Brazilian Amazon. Our findings underscore that the success of this lineage is intrinsically linked to a remarkably complex, modular extrachromosomal landscape, as well as to its high degree of clonal stability. The recovery of the ST-15 lineage from diverse clinical sources, including tracheal aspirates, urine, and blood, highlights its remarkable versatility and potential for systemic dissemination within the hospital environment. The reconstruction of the isolate’s mobilome through hybrid sequencing revealed that resistance is not hosted on a single large element, but rather distributed across multiple small and highly stable plasmids. The high-resolution characterization of these replicons, such as pAB_OXA58_IEC02_2, demonstrated a dense concentration of antibiotic resistance genes (ARGs) within compact scaffolds, where the association of *bla*_OXA−58_ with the IS*Aba*3 element and pdif modules ensures both mobility and persistence. Furthermore, the identification of diverse toxin-antitoxin systems (e.g., AbkAB, RelE/ParE) and virulence factors (e.g., TonB, EsiB) co-localized with resistance determinants highlights a functional clustering that minimizes metabolic burden while improving efficiency and survival. Another important finding was the association of *bla*_OXA−23_ with two other IS elements in a new gene synteny.

The evolution of the ST-15 lineage from a sporadically reported sequence type to a robust MDR pathogen carrying a new repertoire of chromosomal and plasmid-borne carbapenemases, poses a significant threat to local and global healthcare systems. These results emphasize the urgent need for continuous genomic surveillance in the Amazon region and the implementation of hybrid assembly strategies to accurately resolve the mosaic architecture of these extrachromosomal resistance platforms. This approach is essential for anticipating the trajectory of future hospital outbreaks and developing effective strategies to mitigate the dissemination of high-risk *A. baumannii* clones.

## Supplementary Information

Below is the link to the electronic supplementary material.

Supplementary Material 1

## Data Availability

The whole genome shotgun project has been deposited at DDBJ/ENA/GenBank under the accession number JBRYIW000000000. The version described in this paper is JBRYIW010000000.
