# Supplementary material for: Outbreak of Multidrug-Resistant (MDR) Acinetobacter baumannii ST-15 Strains Co-producing OXA-23 and OXA-58 in South America
Source: Curr Microbiol. 2026 Oct 6;83(11):587. doi: 10.1007/s00284-026-05160-1 (PMC13642431; doi:10.1007/s00284-026-05160-1)
Supplement: Supplementary file 1 [file 284_2026_5160_MOESM1_ESM.docx]

**SUPPLEMENTARY MATERIAL**

**First description of Multidrug-Resistant (MDR) *Acinetobacter baumannii* ST-15 strains co producing OXA-23 and OXA-58 in South America**

Aline Madeira Marques Saraiva^1^, Amália Raiana Fonseca Lobato^2^, Maria Paula Cruz Schneider^3^, Diego Assis das Graças^1^, Adonney Allan de Oliveira Veras^1^, Artur Silva^1^, Danielle Murici Brasiliense^2^, Rafael Azevedo Baraúna^1*^

**Figure S1.** **A. Bioinformatics workflow for genomic analysis of *Acinetobacter baumannii* AB_OXA58_IEC07.** The stages comprise: (1) Genomic sequencing on a long-read platform (Oxford Nanopore); (2) Primary data processing, including quality control and read filtering (NanoPlot and NanoFilt); (3) Genome reconstruction by *de novo* assembly (Flye) and completeness validation (BUSCO); and (4) Structured functional annotation via the Prokka pipeline, utilizing gene prediction algorithms and protein profile searches based on Hidden Markov Models (HMM).

**A.**

**B. Bioinformatics workflow for genomic analysis of the *Acinetobacter baumannii* AB_OXA58_IEC02 plasmid**. (1) Sequencing on the PromethION 2 Solo platform; (2) Raw data processing; (3) Assembly using SPAdes v.4.2.0 and Unicycler; (4) Resistome Analysis using staramr and CARD database; (5) Characterization via MOB-suite v.3.1.9 and (6) Confirmation by BLASTn against GenBank (≥90% identity and ≥60% coverage).

**Table S1.** Resistance profile of the eight *Acinetobacter baumannii* isolates is shown below. Three strains were sensitive to ampicillin+sulbactam (AB_OXA58_IEC03, AB_OXA58_IEC05, and AB_OXA58_IEC06), one strain was sensitive to amicacin (AB_OXA58_IEC08), and six strains were sensitive to tetracycline (AB_OXA58_IEC01, AB_OXA58_IEC02, AB_OXA58_IEC03, AB_OXA58_IEC04, AB_OXA58_IEC05 and AB_OXA58_IEC06).

| Sample ID | PIP | | APS | | PTZ | | TCC | | CAZ | | FEP | | CTX | | CRO | | IPM | | GEN | | TOB | | AMI | | TET | | CIP | |
| --- | --- | --- | --- | --- | --- | --- | --- | --- | --- | --- | --- | --- | --- | --- | --- | --- | --- | --- | --- | --- | --- | --- | --- | --- | --- | --- | --- | --- |
|  | **H** | **P** | **H** | **P** | **H** | **P** | **H** | **P** | **H** | **P** | **H** | **P** | **H** | **P** | **H** | **P** | **H** | **P** | **H** | **P** | **H** | **P** | **H** | **P** | **H** | **P** | **H** | **P** |
| IEC01 | 0 | R | 16 | S | 10 | R | 0 | R | 0 | R | 13 | R | 0 | R | 0 | R | 10 | R | 0 | R | 8 | R | 8 | R | 19 | S | 0 | R |
| IEC02 | 0 | R | 13 | I | 12 | R | 0 | R | 0 | R | 12 | R | 0 | R | 0 | R | 8 | R | 0 | R | 10 | R | 0 | R | 19 | S | 0 | R |
| IEC03 | 0 | R | 15 | S | 12 | R | 0 | R | 0 | R | 10 | R | 0 | R | 0 | R | 9 | R | 10 | R | 12* | R | 0 | R | 20 | S | 0 | R |
| IEC04 | 0 | R | 8 | R | 0 | R | 0 | R | 0 | R | 0 | R | 0 | R | 0 | R | 10 | R | 11 | R | 12 | R | 10 | R | 20 | S | 0 | R |
| IEC05 | 8 | R | 16 | S | 15 | R | 0 | R | 0 | R | 15 | I | 0 | R | 0 | R | 10 | R | 0 | R | 8 | R | 9 | R | 16 | S | 0 | R |
| IEC06 | 8 | R | 16 | S | 12 | R | 0 | R | 0 | R | 14 | R | 0 | R | 0 | R | 10 | R | 0 | R | 10* | R | 11 | R | 21 | S | 0 | R |
| IEC07 | 0 | R | 8 | R | 13 | R | 0 | R | 0 | R | 12 | R | 0 | R | 0 | R | 10 | R | 0 | R | 10 | R | 9 | R | 9 | R | 0 | R |
| IEC08 | 0 | R | 10 | R | 12 | R | 0 | R | 0 | R | 11 | R | 0 | R | 0 | R | 10* | R | 0 | R | 12 | R | 17 | S | 9 | R | 0 | R |

Legend: H: Halo (mm); P: Profile of Resistance; R: Resistant; S: Susceptible; I: Intermediate; Piperacilin: PIP; Ampicilin/ Sulbactam: APS; Piperacilin/Tazobactam: PIP/TAZ; Ticarcillin/ Clavulanate: TCC; Ceftazidime: CAZ; Cefepime: FEP; Cefotaxime: CTX; Ceftriaxone: CRO; Imipenem: IPM; Gentamicin: GEN; Tobramicin: TOB; Amikacin: AMI; Tetraciclin: TET; Ciprofloxacin: CIP

**Table S2.** Description of the Insertion Sequences of *Acinetobacter baumannii* (IS*Aba*) detected by ISFinder presente in the genome of *A. baumannii* AB_OXA58_IEC07. These IS elements are responsible for the mobilization and overexpression of the *bla* genes. IS*Aba1* and IS*Aba125* detected in the Contig_1, regulate the expression of *ampC* and *bla*_OXA-51_, respectively. In *A. baumannii* AB_OXA58_IEC07, both the *bla*_OXA-23_ and *bla*_ADC-181_genes were located a few base pairs upstream of the IS*Aba1* and IS*Aba33*. The IS*Aba42* appears conected to the *bla*_OXA-51_ gene, representing an unusual genetic context.

| Sequences producing/ significant alignments | IS Family | Group | Score (bits) | e- value |
| --- | --- | --- | --- | --- |
| Contig_1 | | | | |
| IS*Aba1* | IS4 | IS10 | 2284 | 0.0 |
| IS*Aba31* | IS5 | IS427 | 1598 | 0.0 |
| IS*Aba33* | IS4 | IS10 | 1247 | 0.0 |
| IS*Aba42* | IS256 |  | 2617 | 0.0 |
| IS*Aba52* | IS6 |  | 1019 | 0.0 |
| IS*Aba125* | IS30 |  | 2147 | 0.0 |
| Contig_6 | | | | |
| IS*Aba5* | IS5 | IS903 | 714 | 0.0 |
| IS*Aba12* | IS5 | IS903 | 944 | 0.0 |
| IS*Aba13* | IS5 | IS903 | 579 | 1e-163 |
| IS*Aba40* | IS5 | IS903 | 515 | 1e-144 |
| IS*Aba52* | IS6 |  | 1023 | 0.0 |
| IS*Aba53* | IS5 | IS903 | 664 | 0.0 |
| IS*Aba59* | IS5 | IS903 | 809 | 0.0 |
| IS*Aba62* | IS5 | IS903 | 803 | 0.0 |
| IS*Aba68* | IS5 | IS903 | 771 | 0.0 |
| Contig_8 | | | | |
| IS*Aba16* | IS66 |  | 192 | 8e-47 |
| IS*Aba17* | IS66 |  | 58 | 8e-07 |
| IS*Aba25* | IS66 |  | 167 | 1e-30 |
| IS*Aba46* | IS66 |  | 198 | 2e-47 |
| IS*Aba49* | IS66 |  | 121 | 6e-26 |
| IS*Aba52* | IS6 |  | 137 | 1e-30 |

**Table S3.** Gene content and functional annotation of plasmid pAB_OXA58_IEC07_1 (pAC161-like), recovered from contig_8 of *A. baumannii* isolate AB_OXA58_IEC07. The pAC161 reference plasmid (rep_cluster_1226 and rep_cluster_1282; secondary cluster ID AL237) is a mobilizable, incomplete plasmid of 25.4 kb and harbors a MOB_Q_-type relaxase.

| Locus/Gene | Protein Product | Coordinates (Start-End) | Orientation | Functional Category |
| --- | --- | --- | --- | --- |
| *esiB* | Virulence factor EsiB (SEL1/TPR domains) | 155 – 673 | Forward | Virulence / Host Evasion |
| *repM-1* | Replication initiation protein RepM | 2324 – 3253 | Forward | Replication (RepAci4) |
| *repB-1* | Replication regulatory protein RepB | 3332 – 3856 | Forward | Replication Control |
| *IS6-like* | IS6-family transposase | 4200 – 4904 | Forward | DNA Mobilization (IS) |
| *higB2* | Toxin HigB | 5738 – 5968 | Foward | Plasmid Stability (TA System) |
| *tonB* | TonB-dependent siderophore receptor | 7683 – 10094 | Reverse | Nutrient/Iron Acquisition |
| *brnT/A* | Toxin- Antitoxin BrnT/ BrnA | 10222 – 10810 | Reverse | Plasmid Stability (TA System) |
| *IS6-like* | IS6-family transposase | 11346 – 12050 | Reverse | DNA Mobilization (IS) |
| *aac(3)* | Aminoglycoside 3-N-acetyltransferase | 12097 – 12915 | Reverse | Antibiotic Resistance |
| *bla*_TEM-1_ | Beta-lactamase TEM-1 | 13057 – 13917 | Reverse | Antibiotic Resistance |
| *serine recombinase* | DNA-invertase Bin3 (Tn552-associated) | 15826 – 16437 | Forward | DNA Recombination |
| *mph(E)* | Macrolide 2'-phosphotransferase | 16627 – 17511 | Reverse | Antibiotic Resistance |
| *msr(E)* | ABC transporter (macrolide efflux) | 17567 – 19042 | Reverse | Antibiotic Resistance |
| *higA2* | Toxin - Antitoxin HigA-2/B-2 | 19534 – 20119 | Reverse | Plasmid Stability (TA System) |
| *repM-2* | Replication initiation protein RepM | 20936 – 21469 | Reverse | Replication (RepAci4) |
| *repB-2* | Replication regulatory protein RepB | 21554 – 22477 | Reverse | Replication Control |
| *mobQ* | Mobilization protein MobQ (Relaxase) | 23052 – 24488 | Reverse | Plasmid Mobilization |
| *mobS* | Mobilization protein MobS | 24659 – 24964 | Foward | Plasmid Mobilization |

Note: SEL1 (Suppressor of lin-12-like) and TPR (Tetratricopeptide Repeat)

**Figure S2-A. Structural map of the pAC163-like plasmid reconstructed via SPAdes (Isolate AB_OXA58_IEC02).** Replicon of the 2.3 kb, this assembly identify of the *mobQ* relaxase and the resistance genes *bla*_TEM-1_ and *aac (3).*

Note: The grey inner ring represents the reference backbone of pAC163, showing high structural conservation despite the fragmented nature of the initial short-read assembly.

**Figure S2-B. Comparative synteny of the large carbapenemase-carrier plasmid against pDETAB5.** Circular map of the 89.8 kb plasmid carrying *bla*_OXA-58_. This specific assembly showed 100% identity with the reference plasmid pDETAB5, providing a critical benchmark for validating the mobilization unit (IS*Aba3 - bla*_OXA-58_-IS*Aba3*). Note the presence of the XerC/D site-specific recombinases (red arrows) and additional resistance determinants like *aac (3) -IIa*.

**Figure S3. Molecular confirmation of *bla*_OXA-58_ in purified plasmid DNA.** Agarose gel electrophoresis (1.5%) of PCR products amplified from plasmid DNA extractions. The bands confirm the presence of the *bla*_OXA-58_ gene in the plasmid pool for the isolates AB_OXA58_IEC01, AB_OXA58_IEC02, AB_OXA58_IEC03. The detection of the intrinsic *bla*_OXA-51_ gene was used to confirm the *A. baumannii* identity of the source material

Legenda: **(+)** OXA-58 / (+) OXA-51: Positive controls; **(-)**: Negative control; Ladder: 1kb molecular weight marker

**Table S4.** Gene content and functional annotation of plasmids recovered from *A. baumannii* isolate AB_OXA58_IEC02. Plasmids were reconstructed from assembled contigs and manually curated to define replicon type, mobilization modules, and accessory gene content

**A. Plasmid pAB_OXA58_IEC02_1**: A ~2.6 kb plasmid belonging to the RepAci1 group. It encodes a RepM replication protein, the AbkB/A toxin–antitoxin system, TonB-dependent receptor, septicicolystin-like bacteriocin gene and oriT site, suggesting potential roles in plasmid maintenance, nutrient acquisition, and competition

| Locus/Gene | Protein Product | Coordinates (Approx.) | Orientation | Functional Category |
| --- | --- | --- | --- | --- |
| *repAci1* | Replication initiation protein | 1 – 951 | Forward | Replication |
| *repM* | Replication initiation protein RepM | 944 - 1519 | Forward | Replication |
| *Xer C/D* | Pdif-site | 1827 – 1854 | Foward | Recombination |
| *sel1* | Repeat Family protein | 1871 – 2413 | Forward | Replication |
| *Xer D/C* | Pdif-site | 2527 – 2554 | Foward | Recombination |
| *abkB/abkA* | Toxin-Antitoxin System | 2753 – 3350 | Reverse | Plasmid Stability |
| *tonB* | TonB-dependent receptor | 3478 – 5889 | Foward | Nutrient Acquisition |
| *septicicolystin-like* | Septicicolystin-like bacteriocin | 6194 – 6655 | Forward | Bacterial Competition |
| *Xer D/C* | Pdif-site | 6945-6972 | Foward | Recombination |
| *aac(3)-IIa* | Aminoglycoside 3-N-acetyltransferase | 6978 – 7151 | Reverse | Antibiotic Resistance |
| *aac(3)* | Aminoglycoside acetyltransferase | 7151 – 7522 | Reverse | Antibiotic Resistance |
| *oriT* | Origin of Transfer (site) | 7844 – 7916 | Foward | Plasmid Mobilization |
| *repAci iterons (1, 2, 3,4)* | Rep-binding repeated | 8591 – 8678 | Foward | Replication Regulation |

**B.** **Plasmid pAB_OXA58_IEC02_2:** A multidrug-resistance plasmid of approximately 1.7 kb, characterized as AD745-like replicon. It carries the *bla*_OXA-58_ carbapenemase gene flanked by IS*Aba3* and IS6-like elements, and includes a MobA/L-type mobilization module and the BrnTA toxin–antitoxin stability system.

| Locus/Gene | Protein Product | Coordinates (Approx.) | Orientation | Functional Category |
| --- | --- | --- | --- | --- |
| *repM* | Replication initiation protein RepM | 1009 – 1542 | Forward | Replication |
| *pRiA4b* | Plasmid replication-associated protein | 1829 – 2356 | Forward | Replication |
| *Type II TA* | Type II Toxin-Antitoxin System | 4524 – 4844 | Forward | Plasmid Stability |
| *lsyE* | Lysine exporter LysE | 5583 – 6188 | Reverse | Regulation |
| *araC* | AraC-family transcriptional regulator | 6238 – 7065 | Forward | Transporter / Efflux |
| IS*Aba3* | family transposase | 7193 – 7945 | Reverse | DNA Mobilization |
| *bla*_OXA-58_ | Carbapenemase | 8036 – 8878 | Reverse | Antibiotic Resistance |
| *IS6-like* | IS6-family transposase | 9083 – 9787 | Forward | DNA Mobilization |
| *brnT/brnA* | Toxin-Antitoxin System | 10323 – 10911 | Forward | Plasmid Stability |
| *znuD2* | TonB-dependent ZnuD2 receptor | 11039 – 13450 | Forward | Nutrient Acquisition |
| *dip1984* | Dipeptide ABC transporter ATP-binding protein (DppF-like) | 13755 – 14216 | Forward | Transporter/ ABC transport |
| *mobA/mobL* | Mobilization protein MobA/L | 15458 – 16894 | Forward | Plasmid Mobilization |

**C. Plasmid pAB_OXA58_IEC02_3:** A 1.7 kb mobilizable, cryptic plasmid with a multireplicon structure (*repM* and *pRiA4b*). No acquired antimicrobial-resistance genes were identified; however, it encodes a type II toxin–antitoxin system and a MobA/MobL mobilization module.

| Locus/Gene | Protein Product | Coordinates (Approx.) | Orientation | Functional Category |
| --- | --- | --- | --- | --- |
| *Type II TA* | Type II Toxin-Antitoxin System | 5857 – 6177 | Reverse | Plasmid Stability |
| *pRiA4b* | Plasmid replication-associated protein | 8345 – 8872 | Reverse | Replication |
| *repM* | Replication initiation protein RepM | 9721 – 10602 | Reverse | Replication |
| *mobA/mobL* | Mobilization protein MobA/L | 11150 – 12319 | Reverse | Plasmid Mobilization |

**D. Plasmid pAB_OXA58_IEC02_4:** A 1.44 kb mobilizable plasmid carrying the tetracycline-resistance determinant *tet (39)* and its transcriptional regulator *tetR*. The replicon is characterized by a RepM protein and the plasmid harbors a MOB_Q_-type relaxase.

| Locus/Gene | Protein Product | Coordinates (Approx.) | Orientation | Functional Category |
| --- | --- | --- | --- | --- |
| *tet39* | Tetracycline resistance protein Tet39 | 0 – 866/  5689 – 6010 | Forward | Antibiotic Resistance |
| *tetR* | Tetracycline transcriptional regulator | 932 – 1573 | Forward | Regulation |
| *mobQ* | Mobilization protein MOB_Q_ | 2262 – 3233 | Reverse | Plasmid Mobilization |
| *repM* | Replication initiation protein RepM | 4016 – 4939 | Reverse | Replication |
